# Exosome Treatment Enhances Anti-Inflammatory M2 Macrophages and Reduces Inflammation-Induced Pyroptosis in Doxorubicin-Induced Cardiomyopathy

**DOI:** 10.3390/cells8101224

**Published:** 2019-10-09

**Authors:** Dinender K. Singla, Taylor A. Johnson, Zahra Tavakoli Dargani

**Affiliations:** Division of Metabolic and Cardiovascular Sciences, Burnett School of Biomedical Sciences, College of Medicine, University of Central Florida, Orlando, FL 32816, USAzahra.tavakoli@knights.ucf.edu (Z.T.D.)

**Keywords:** doxorubicin, cardiotoxicity, pyroptosis, inflammation, embryonic stem cells, exosomes

## Abstract

Doxorubicin (Dox) is an effective antineoplastic agent used to treat cancers, but its use is limited as Dox induces adverse cardiotoxic effects. Dox-induced cardiotoxicity (DIC) can lead to heart failure and death. There is no study that investigates whether embryonic stem cell-derived exosomes (ES-Exos) in DIC can attenuate inflammation-induced pyroptosis, pro-inflammatory M1 macrophages, inflammatory cell signaling, and adverse cardiac remodeling. For this purpose, we transplanted ES-Exos and compared with ES-cells (ESCs) to examine pyroptosis, inflammation, cell signaling, adverse cardiac remodeling, and their influence on DIC induced cardiac dysfunction. Therefore, we used C57BL/6J mice ages 10 ± 2 weeks and divided them into four groups (*n* = 6–8/group): Control, Dox, Dox + ESCs, and Dox + ES-Exos. Our data shows that the Dox treatment significantly increased expression of inflammasome markers (TLR4 and NLRP3), pyroptotic markers (caspase-1, IL1-β, and IL-18), cell signaling proteins (MyD88, p-P38, and p-JNK), pro-inflammatory M1 macrophages, and TNF-α cytokine. This increased pyroptosis, inflammation, and cell signaling proteins were inhibited with ES-Exos or ESCs. Moreover, ES-Exos or ESCs increased M2 macrophages and anti-inflammatory cytokine, IL-10. Additionally, ES-Exos or ESCs treatment inhibited significantly cytoplasmic vacuolization, myofibril loss, hypertrophy, and improved heart function. In conclusion, for the first time we demonstrated that Dox-induced pyroptosis and cardiac remodeling are ameliorated by ES-Exos or ESCs.

## 1. Introduction

Doxorubicin (Dox) is a secondary metabolite of *Streptomyces peucetius* var. *caesius*, a family of anthracyclines. This powerful drug is an antineoplastic therapeutic agent used to treat a wide variety of solid organ tumors and hematologic malignancies, including leukemia, lymphoma, breast cancer, and lung cancer in adults and pediatric patients [1,2,3]. However, medicinal use of this drug is limited due to acute and chronic side effects such as alopecia, vomiting, nausea, cardiac dysfunction, and heart failure [4,5]. Dox-induced cardiotoxicity (DIC) depends on the drug dose; however, it leads to complex and time-dependent cardiomyopathy that develops cardiac pathophysiological and cellular changes. Cardiomyocyte apoptosis, fibrosis, hypertrophy, and cellular vacuolization are reported by various investigators [6,7,8]. Apoptosis has also been reported in non-cardiac myocytes such as endothelial cells [9,10]. Recent studies suggest that DIC may involve infiltration of M1 macrophages that induces inflammation and pathogenesis [1,11]. However, this remains unknown whether presence of inflammation induces inflammation-mediated cell death—termed pyroptosis in DIC.

So far, oxidative stress has been considered as a major prognostic factor in DIC progression. Therefore, antioxidant therapeutic options were adapted to block these adverse cardiac events in response to Dox-induced cell death, remodeling, and decrease in cardiac function [6,12,13,14,15,16,17,18] with limited success. However, alternative strategies are still needed to impede these detrimental effects to the heart following Dox treatment.

Stem cells, particularly embryonic stem cells (ESCs), have been widely accepted as an effective therapeutic approach in many diseases, including cardiovascular disorders [8,19]. Albeit promising reports of success, these undifferentiated pluripotent cells have few fundamental issues such as the formation of teratomas, which is considered the major obstacle blocking ESCs from being considered for human use [20,21]. Moreover, following transplantation of ESCs, only a limited number of cells can engraft and differentiate into heart cells or participate in the inhibition of adverse cardiac remodeling [22,23]. Therefore, recent studies have focused on embryonic stem cell-derived exosomes (ES-Exos) as a safer alternative approach to ESC therapy [23,24,25]. Exosomes (Exos) are cell-derived vesicles, which contain proteins, lipids, growth factors, miRNAs, and anti-inflammatory cytokines that are released through exocytosis [1,26,27]. Recent literature suggests that ES-Exos have the potential to repair infarcted heart [28]. However, this remains to be established in the Dox-induced cardiotoxicity.

To the best of our knowledge, we report for the first time the following major findings of this study: 1) DIC promotes an inflammasome formation (TLR4 and NLRP3 proteins) that leads to inflammation-induced novel form of cell death, pyroptosis (caspase-1, IL1-β, and IL-18), 2) ES-Exos inhibit pyroptosis in a similar potential and capability as observed with ESCs, 3) Exos treatment convert pro-inflammatory M1 macrophages into anti-inflammatory M2 macrophages that attenuates pyroptosis in DIC, and 4) ES-Exos inhibit adverse cardiac remodeling and improve cardiac function.

## 2. Materials and Methods

### 2.1. Cell Culture and Exosomes Preparation

ES cells (CGR8, a mouse ES cell line) were purchased from American Type Culture Collection (ATCC, Manassas, VA, USA) and cultured as previously described [1,29]. In brief, ESCs were cultured in 100 mm^2^ tissue culture dishes (ThermoFisher Scientific Waltham, MA, USA) and grown with Dulbecco’s modified Eagle’s medium (DMEM; ThermoFisher Scientific; cat# 11965092) supplemented with 15% ES fetal bovine serum (Gibco; cat# 16141079, Gaithersburg, MD, USA), leukemia inhibitory factor (Millipore Sigma; cat# ESG1107, Burlington, MA, USA), glutamine (ThermoFisher Scientific; cat# 25030081), penicillin/streptomycin (P/S; ThermoFisher Scientific; cat# 15070063), sodium pyruvate (ThermoFisher Scientific; cat# 11360070), and β-mercaptoethanol (ThermoFisher Scientific; cat# 21985023). After 48 h, cell culture medium was replaced with serum-free knockout DMEM (ThermoFisher Scientific; cat# 10829018) supplemented with P/S. 48 h post-media exchange, exosomes were isolated using the Exoquick TC exosome isolation kit (SBI; cat# EXOTC50A-1, Palo Alto, CA, USA) according to the manufacturer’s instructions. In brief, the medium was collected from cell culture plates and centrifuged at 1500 rpm for 15 min to remove cell debris, and then the supernatant was transferred to a sterile tube. The collected supernatant was mixed with Exoquick-TC exosome precipitation solution (SBI) in a 5:1 ratio for 12 h at 4 °C according to the manufacturer’s protocol. Following this, the mixture was centrifuged at 1500× *g* for 1 h, and after aspirating the supernatant, the exosome pellet was obtained and stored at –80 °C for further use. Exos characterization using exosome specific markers HSP-70 and CD63 is recently reported by us [1].

### 2.2. Animal Preparation

All animal protocols were approved by University of Central Florida Institutional Animal Care and Use Committee (IACUC). C57BL/6J mice (10 ± 2 weeks of age, male and female; JAX: 000664) were assigned into four groups: Control (injected with normal saline), Dox, Dox + ESCs, and Dox + ES-Exos. There were *n* = 6–8 animals in each group.

**Figure 1:**
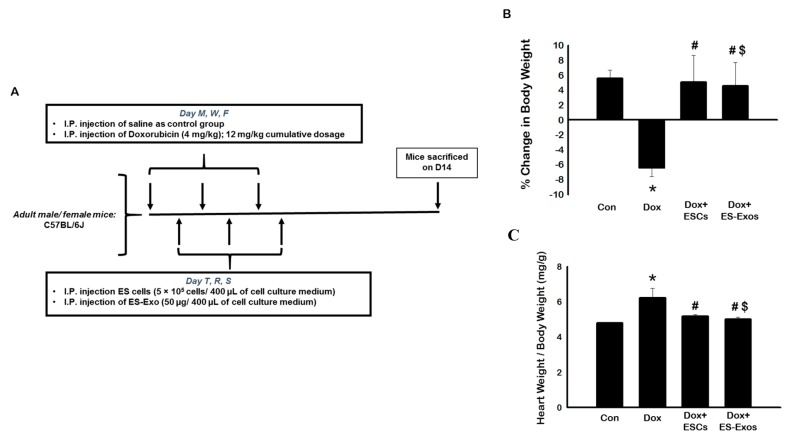
Embryonic stem cells (ESCs) or the embryonic stem cell-derived exosome (ES-Exos) treatment improve healthy weight gain in mice. (**A**) Study design and schematic schedule for injections. (**B**) Bar graphs showing the percentage of change in BW (body weight) over the 14 days trial. (**C**) Bar graph demonstrating the ratio of heart weight-to-body weight (in *mg*/*g*) on D14. Error bars = mean ± S.E.M. * *p* < 0.05 vs. control, ^#^ *p* < 0.05 vs. Dox, ^$^ *p* = non significant (NS) Dox + ES-Exos vs. Dox + ESCs one way ANOVA followed by a Tukey test. Scale bar = 100 µm, *n* = 6–7. Abbreviation: M, Monday; T, Tuesday; W, Wednesday; R, Thursday; F, Friday; S, Saturday; Dox, doxorubicin; ESCs, embryonic stem cells; ES-Exos, embryonic stem cell-derived exosomes.

Dox was injected in three intraperitoneal (i.p) injections (4 mg/kg body weight), on alternative days in a week time span (Monday, Wednesday, and Friday) for a cumulative dose of 12 mg/kg (Figure 1A). For treatment groups, ESCs (5 × 10^5^ ESCs in 400 µL medium/injection) or ES-Exos (400 µL/injection with 50 µg concentration of ES-Exos) were injected on alternative days between Dox treatments (Tuesday, Thursday, and Saturday; Figure 1A). Body weight (BW) was measured before starting injections as well as at the time of sacrifice. Two weeks following the last injection, mice were sacrificed under sedation with 4% isoflurane via nose cone and subsequent cervical dislocation. Hearts were harvested, washed in phosphate buffered saline (PBS), weighed, and divided into two halves. The top portion was saved in RNA later for western blot analysis and RNA isolation, and the bottom portion was placed in 4% paraformaldehyde (Fisher Scientific) for immunohistochemistry staining.

### 2.3. Immunohistochemistry (IHC) Staining

The bottom half of the heart tissue preserved in paraformaldehyde was embedded in paraffin and serial sectioned at 5 µm thickness. Sections were placed on microscope slides and double immunohistochemistry (IHC) staining was performed, as previously described [6]. In brief, sections were deparaffinized, rehydrated, and then blocked with 10% normal goat serum blocking reagent (Vector Lab; cat# S-1000, Burlingame, CA, USA) for one hour at room temperature (RT) prior to incubation with primary antibodies for inflammasome markers; TLR4 (1:50; Abcam; cat# ab13556, Cambridge, MA, USA) and NLRP3 (1:50; Abcam; cat# ab214185), for pyroptotic markers; caspase-1 (1:50; Abcam; cat# ab138483), IL-1β (1:50; Abcam; cat# ab9722), and IL-18 (1:50; Abcam; cat# ab71495), for pro-inflammatory cytokine, TNF-α (1:50; Abcam; cat# 6671), for marker of M1 macrophages, induced nitric oxide synthase (iNOS; 1:50; Abcam; cat# 15323), for M2 macrophages marker, CD206 (1:50; Abcam; cat# 4693) and for anti-inflammatory cytokine, IL-10 (1:50; Abcam; cat# ab33471) overnight at 4 °C. Following primary incubation, sections were incubated with a secondary antibody, Alexa 568 goat anti-rabbit (1:50; Invitrogen; cat# A-11011, Carlsbad, CA, USA) or Alexa 594 goat anti-rat (1:50; Invitrogen; cat#A-11007). Anti-α-sarcomeric actin antibody (1:30 dilution; Sigma-Aldrich; cat# 2172, Burlington, MA, USA), which specifically stains for cardiomyocytes, was applied on the sections. Following incubation with biotinylated Anti-Mouse IgG reagent (MOM kit, Vector Lab; cat# FMK-2201) and fluorescein Avidin DCS (green color reaction), slides were covered with mounting medium containing DAPI (4′,6-diamino-2-phenylindole; Vector Lab; Burlingame, CA; cat# NC9524612) and cover slipped. Pictures were taken using Olympus fluorescent (Center Valley, PA, USA) and Leica confocal (Buffalo Grove, IL, USA) microscopes. Quantitative data for pyroptotic cell death, secreted pro-inflammatory cytokine (TNF-α), anti-inflammatory cytokine (IL-10), and macrophages were calculated by dividing total positive cells over total DAPI times 100 ((total cells**^+ve^**/total DAPI)*100). Graphs were created using image J and Sigma Plot software.

### 2.4. SDS-PAGE and Western Blot Analysis

Western blot was performed as previously reported [30]. In brief, the top portion of heart tissue saved in RNA later solution was lysed using the RIPA (radio-immunoprecipitation assay) lysis buffer, the supernatant was collected, and protein concentration was estimated via a Bio-Rad assay. 50 µg of protein was loaded into 10% or 12% sodium dodecyl sulfate (SDS) gels and run at 150 V for 1 h. Gels were transferred onto polyvinylidene difluoride (PVDF; BioRad; cat# 162-0177, Hercules, CA, USA) membranes (Bio-Rad, CA) using a semi-dry transfer machine (Bio-Rad). Membranes were blocked with 5% skim milk prior to incubation with primary antibodies for TLR4 (1:1000; Abcam; cat# ab13556), NLRP3 (1:1000; Cell Signaling; cat# D4D8T, Danvers, MA, USA), caspase-1 (1:1000; Abcam; cat# ab138483), IL-1β (1:1000; Abcam; cat# ab9722), IL-18 (1:1000; Abcam; cat# ab71495), TNF-α (1:500; Abcam; cat# ab6671), IL-10 (1:500; Abcam; cat# ab33471), MyD88 (1:1000; Abcam; cat# ab135693), p-P38 (1:1000; Cell Signaling; cat# 4631S), p-JNK (1:1000; Cell Signaling; cat# 4668S), MMP-9 (1:1000; Abcam; cat# ab38898), and β-actin (1:1000; Cell Signaling; cat# 4967L) overnight at 4 °C or one hour at RT. Following washing the membranes with 1XTBS-T buffer, goat anti-rabbit IgG-HRP (1:1000; Cell Signaling; cat# 7074S) or goat anti-rat IgG-HRP (1:1000; Santa Cruz Biotechnology; cat# sc-2006, Dallas, Texas, USA) secondary antibody was used for one hour at RT. After exposure with enhanced chemiluminescence (ECL) substrates (Fisher Scientific; cat# 32106), membranes were developed on x-ray film. Using captured images, densitometry analysis was performed using image J.

### 2.5. cDNA Synthesis and RT-PCR Reaction

Total RNA from heart tissue was isolated by using a Trizol^TM^ reagent. One µg of RNA was reverse-transcribed into cDNA using the Superscript^TM^ III First Strand Synthesis system (Invitrogen, Carlsbad, CA). cDNA (50 ng) sample used to perform quantitative real time PCR reaction by CFX96 iCycler Multicolor Real-Time PCR Detection System (Bio-Rad, Hercules, CA) with SYBR Green (Invitrogen, Carlsbad, CA). Polymerase chain reaction (PCR) was carried out with Caspase-1, IL-1β, and IL-18 specific primers for mouse targets (Table 1), resulting in 200 bp fragments. As a reference gene, we used GAPDH primers, resulting in a 200-bp fragment. PCR was performed with an initial step of denaturation at 50 °C for 2 min, 95 °C for 10 min followed by 40 cycles of 95 °C for 20s and 60 °C for 20s. Melt curves were established for the reactions. Normalized fold expression was calculated using the 2^−∆∆Ct^ method.

### 2.6. Determination of Cytoplasmic Vacuolization, Myofibrillar Loss, and Cardiac Hypertrophy

Hematoxylin and eosin (H&E) staining was performed for the evaluation of cytoplasmic vacuolization, myofibrillar loss, and cardiac hypertrophy through staining heart sections with hematoxylin (ThermoScientific Fisher; cat# 7211), acid alcohol 1% (Poly Scientific R&D Corp; cat# S104, Bay Shore, NY, USA), bluing (ThermoScientific Fisher; cat# 73011), and eosin (ThermoScientific Fisher; cat# 7111). Three sections/heart were examined, which were graded from 1 to 5, depending on the presence/absence of either cytoplasmic vacuolization or myofibrillar loss. Image J was used to quantify cardiomyocyte size (mm^2^) at 20× magnification captured pictures.

### 2.7. Determination of Intestinal and Vascular Fibrosis

To examine vascular and interstitial fibrosis, Masson’s trichrome staining was performed to visualize fibrosis as previously published [6]. In brief, paraffin embedded heart tissues were cut into 5 μm serial sections and placed on slides. Following deparaffinization and rehydration, sections were incubated with Bouins fixative (45 min at 62.3 °C; Poly Scientific R&D Corp; cat# S129), and then stained with Weigert iron hematoxylin A&B solution (Poly Scientific R&D Corp; cat# S216 BA and S216 BB), Biebrich scarlet acid-fuschin (Poly Scientific R&D Corp; cat# S125), phosphomolybdic/phosphotungstic solution (Fisher; cat# A248-100, and A237-100), aniline blue (Poly Scientific R&D Corp; cat# S116), and 1% glacial acetic acid (Fisher; cat# A491-212). Interstitial fibrosis was quantified by measuring the total blue area per mm^2^; while, vascular fibrosis was determined by measuring vascular fibrosis/vessel area × 100% on captured images at 20× magnification using NIH image J software.

### 2.8. Echocardiography

At day 14 (D14) after the last injection, 2D-echocardiography was performed using a Sonos 5500 ultrasonograph with a 15 MHz transducer (Philips, Andover MA). M-mode images were captured and analyzed as we published previously [6,11]. The left ventricular internal dimension-diastole (LVIDd), left ventricular internal dimension-systole (LVIDs), fractional shortening (FS; (LVIDd – LVIDs)/LVIDd × 100)), left ventricular volume at end diastole (EDV), left ventricular volume at end systole (ESV), and ejection fraction (EF; (EDV– ESV)/EDV × 100)) were determined with and without ESCs or ES-Exos treatment in DIC.

### 2.9. Statistical Analysis

Data was analyzed using one-way analysis of variance (ANOVA) followed by a Tukey test and expressed as a mean ± SEM. The *p*-value < 0.05 denotes statistical significance.

## 3. Results

### 3.1. Effect of ESCs or ES-Exos on Mice Weight Following Dox Administration

To determine the impact of ESCs or ES-Exos on weight gain or weight loss, mice were weighed prior to the treatment as well as at the time of sacrifice (D14). As represented in Figure 1B, a significant (*p* < 0.05) weight loss was observed in Dox administered mice compared with controls. The weight loss was significantly (*p* < 0.05) improved with ESCs or ES-Exos treatment, suggesting that ESCs or ES-Exos attenuate Dox treatment adverse effects on whole body weight.

Ratio of heart weight to body weight was calculated as described previously [11]. Our data shows a significant (*p* < 0.05) increase in heart weight-to-body weight ratio in Dox administered mice as compared to control mice (Figure 1C). Moreover, treatment with ESCs or ES-Exos reduced the heart weight-to-body weight ratio significantly (*p* < 0.05), suggesting that ESCs or ES-Exos provide protective effects against Dox increased heart weight-to-body weight ratio. Furthermore, this data suggests that ES Exos, a subset of ESCs, have equal beneficial effects as compared to ESCs.

### 3.2. Expression of Inflammasome Formation Proteins (TLR4 and NLRP3) after ESCs or ES-Exos Treatment

**Figure 2:**
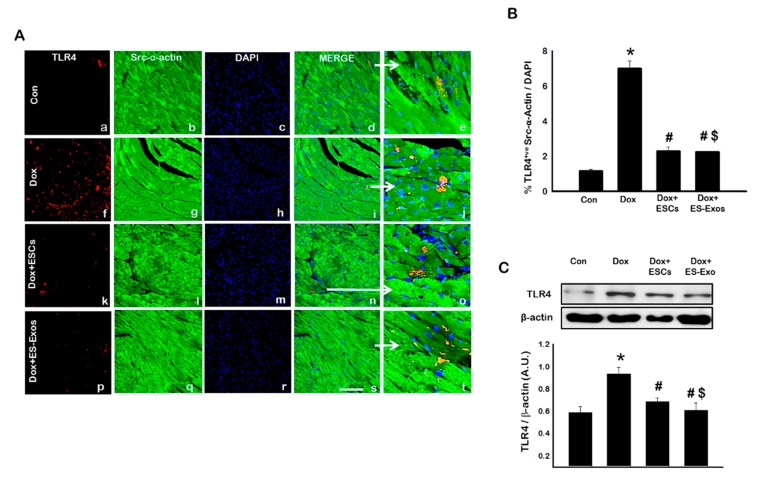
ESCs or ES-Exos administration inhibits TLR4 activation in cardiomyocytes. (**A**) Representative photomicrographs of heart sections stained with src-α-actin and inflammasome marker TLR4. Individual boxes show TLR4^+ve^ cells in red (a, f, k, p), cardiomyocytes in green (b, g, l, q), DAPI in blue (c, h, m, r), merged images (d, i, n, s), and enlarged areas of merged images (e, j, o, t). (**B**) Quantitative analysis-derived histograms of TLR4^+ve^ cardiomyocytes quantified over total DAPI. (**C**) Representative blot and densitometric analysis of TLR4. Error bars = mean ± S.E.M. * *p* < 0.05 vs. control, ^#^ *p* < 0.05 vs. Dox, ^$^ *p* = non significant (NS) Dox + ES-Exos vs. Dox + ESCs, one way ANOVA followed by a Tukey test; western blot quantities are represented as A.U. Scale bar = 100 µm, *n* = 5–6.

**Figure 3:**
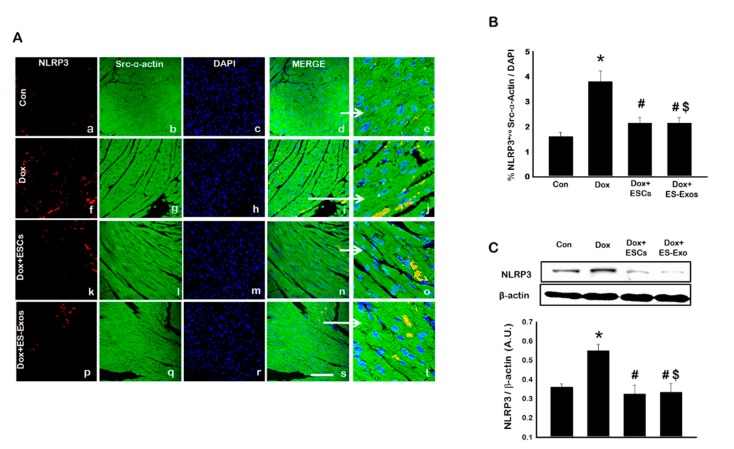
ESCs or ES-Exos treatment inhibits NLRP3 inflammasome formation in the Dox-induced cardiotoxicity (DIC) model. (**A**) Representative photomicrographs of heart sections stained with src-α-actin and inflammasome marker NLRP3. Individual boxes show NLRP3^+ve^ cells in red (a, f, k, p), cardiomyocytes in green (b, g, l, q), DAPI in blue (c, h, m, r), merged images (d, i, n, s), and enlarged areas of merged images (e, j, o, t). (**B**) Quantitative analysis-derived histograms of NLRP3^+ve^ cardiomyocytes quantified over total DAPI. (**C**) Representative blot and densitometric analysis shown for NLRP3. Error bars = mean ± S.E.M. * *p* < 0.05 vs. control, ^#^ *p* < 0.05 vs. Dox, ^$^ *p* = non significant (NS) Dox + ES-Exos vs. Dox + ESCs, one way ANOVA followed by a Tukey test, Western blot quantities are expressed as A.U. Scale bar = 100 µm, *n* = 5–6.

IHC staining was performed to evaluate the expression of TLR4 and NLRP3 in cardiomyocytes following Dox treatment. The presence of TLR4^+ve^ (Figure 2A,B) and NLRP3^+ve^ (Figure 3A,B) cells were significantly (*p* < 0.05) increased in Dox treated group (f–j) compared with control (a–e). However, treatment with ESCs significantly reduced expression of TLR4 and NLRP3 positive cells (k–o). Upon comparing whether ES-Exos had a similar potential to decrease the expression of TLR4 and NLRP3 positive cardiomyocytes, our quantitative data shows ES-Exos significantly (*p* < 0.05) reduced the levels of TLR4 (Figure 2B) as well as NLRP3 (Figure 3B) expression.

To strengthen and confirm our IHC data of TLR4^+ve^ and NLRP3^+ve^ cardiomyocytes, we performed Western blot analysis. Our Western blot data shows a significant increase in TLR4 and NLRP3 compared to control (*p* < 0.05), and when Dox animals were treated with ESCs or ES-Exos, the expression of TLR4 and NLRP3 were significantly reduced (*p* < 0.05; Figure 2C and Figure 3C, respectively). These changes in our Western blot analysis were further confirmed and quantified using densitometric analysis for TLR4 (Figure 2C) and NLRP3 (Figure 3C) markers. This set of TLR4 and NLRP3 data suggests Dox treatment enhanced inflammasome formation, which is attenuated with ESCs or ES-Exos.

### 3.3. Expression of Pyroptotic Markers Caspase-1, IL-1β, and IL-18, after ESCs or ES-Exos Treatment

**Figure 4:**
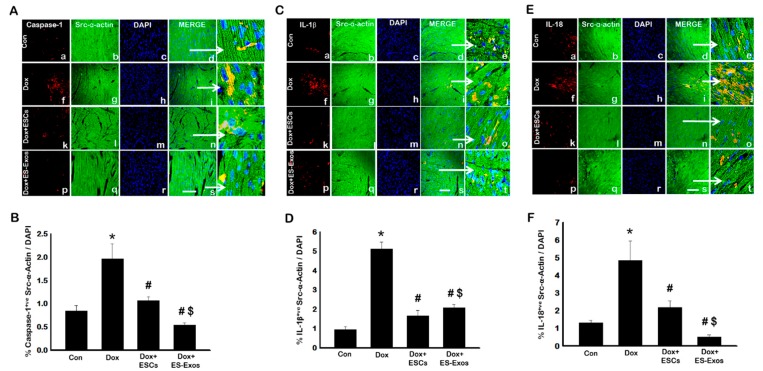
Treatment with ESCs or ES-Exos inhibits Dox-induced pyroptosis within the heart. (**A**) Representative photomicrographs of heart sections stained with src-α-actin and caspase-1. (**C**) Representative confocal microscopy of heart sections stained with src-α-actin and IL-1β. (**E**) Confocal imaging of heart sections co-stained with src-α-actin and IL-18. Individual boxes show Caspase-1^+ve^, IL-β^+ve^, and IL-18^+ve^ cells in red (a, f, k, p), cardiomyocytes in green (b, g, l, q), DAPI in blue (c, h, m, r), merged images (d, i, n, s), and enlarged areas of merged images (e, j, o, t). (**B**,**D**,**F**) Bar graphs derived from quantification of pyroptotic markers, caspase-1, IL-1β, and IL-18, respectively. Error bars = mean ± S.E.M. * *p* < 0.05 vs. control, ^#^ *p* < 0.05 vs. Dox, ^$^ *p* = non significant (NS) Dox + ES-Exos vs. Dox + ESCs, one way ANOVA followed by a Tukey test, scale bar = 100 µm, *n* = 5–6.

To understand whether inflammasome protein formation can initiate pyroptosis, we performed immunofluorescence staining to determine the presence of pyroptotic markers caspase-1, IL-1β, and IL-18 [1,31]. First, our data shows Dox treated animals had significantly higher levels of caspase-1^+ve^ cardiomyocytes compared with control animals (Figure 4A,B, *p* < 0.05). This increased number of positive cells was further decreased with ESCs or ES-Exos treatment. Next, we performed IL-1β staining to determine the effects of ESCs or ES-Exos treatment on IL-1β expression (Figure 4C,D). IHC staining of heart sections revealed a significant increase in the levels of IL-1β in Dox-treated animals compared with control (Figure 4C,D; *p* < 0.05). However, we observed Dox + ESCs or Dox + ES-Exos treated heart sections showed a significant decrease in the number of IL-1β^+ve^ cells compared with Dox treated sections (*p* < 0.05). A third pyroptotic marker, IL-18, was stained to further confirm pyroptotic cell death in Dox treated hearts. Our IHC data showed a significant increase in IL-18^+ve^ cells in Dox treated hearts compared with control (Figure 4E,F; *p* < 0.05). Noticeably, the animal groups that received ESCs or ES-Exos treatment showed significantly reduced numbers of IL-18^+ve^ cells compared with Dox treated hearts (Figure 4F; *p* < 0.05).

**Figure 5:**
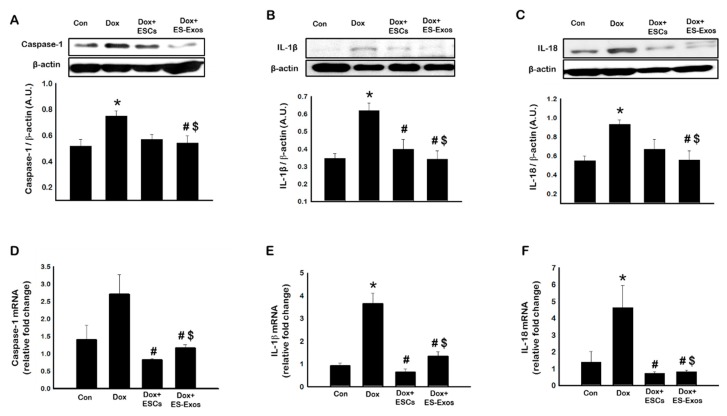
ESCs or ES-Exos decrease pyroptotic markers expression following DIC. Representative blots and densitometric analysis for (**A**) Caspase-1, (**B**) IL-1β, and (**C**) IL-18. Quantitative RT-PCR data for (**D**) caspase-1, (**E**) IL-1β, and (**F**) IL-18. Error bars = mean ± S.E.M. * *p* < 0.05 vs. control, ^#^ *p* < 0.05 vs. Dox, ^$^ *p* = non significant (NS) Dox + ES-Exos vs. Dox + ESCs, one way ANOVA followed by a Tukey test, Western blot quantities are expressed as A.U. *n* = 5–6 for Western blot, *n* = 3–4 for RT-PCR.

To corroborate our immunofluorescence data, we performed Western blot analysis for caspase-1, IL-1β, and IL-18 with and without ESCs or ES-Exos administration (Figure 5). A significant increase in pyroptotic markers caspase-1 (Figure 5A), IL-1β (Figure 5B), and IL-18 (Figure 5C) was observed in Dox-treated animals compared with control (*p* < 0.05). The significant increase in expression of these pyroptotic markers was attenuated with ESCs or ES-Exos treatment (*p* < 0.05). Moreover, Western blot data was further confirmed through gene expression analysis for caspase-1 (Figure 5D), IL-1β (Figure 5E), and IL-18 (Figure 5F) using RT-PCR. Our data indicated a significant (*p* < 0.05) increase in gene expression following Dox administration in IL-1β (Figure 5E) and IL-18 (Figure 5F). An increased trend of caspase-1 was observed but was not statistically significant. Moreover, this increase in these gene expressions was significantly (*p* < 0.05) reduced upon treatment with ESCs or ES-Exos. Collectively, these results demonstrate significantly lower expression of pyroptotic markers caspase-1, IL-1β, and IL-18, suggesting ES-Exos contain specific factors capable of reducing pyroptosis in cardiomyocytes in vivo.

### 3.4. ES-Exos Reduces Pro-Inflammatory M1 Macrophages and Enhances Anti-Inflammatory M2 Macrophages

**Figure 6:**
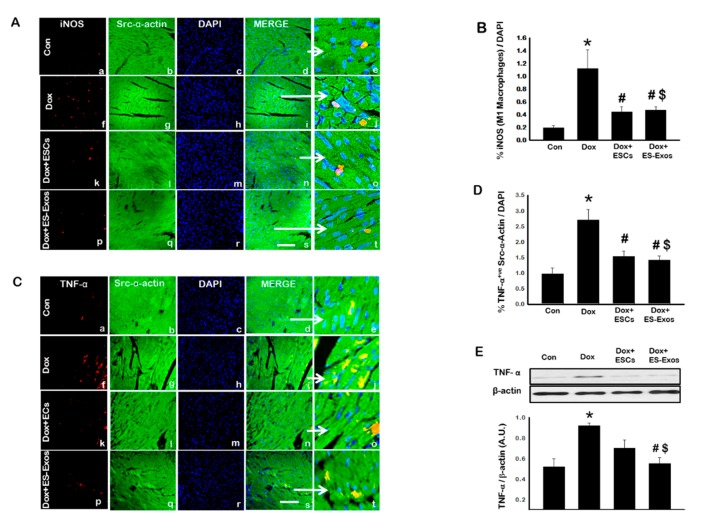
ESCs or ES-Exos treatment reduces M1 macrophages abundance as well as TNF-α secretion. (**A**) Representative photomicrographs of heart sections stained with src-α-actin and iNOS, (**C**) confocal microscopy images for TNF-α. Individual boxes show iNOS^+ve^ and TNF-α^+ve^ cells in red (a, f, k, p), cardiomyocytes in green (b, g, l, q), DAPI in blue (c, h, m, r), merged images (d, i, n, s), and enlarged areas of merged images (e, j, o, t). (**B**) Quantitative analysis-derived histograms show positive cardiomyocytes for iNOS. (**D**) Bar graph resulted from quantitative analysis of TNF-α (**D**). (**E**) Representative blot and densitometric analysis shown for TNF-α. Error bars = mean ± S.E.M. * *p* < 0.05 vs. control, ^#^ *p* < 0.05 vs. Dox, ^$^ *p* = non significant (NS) Dox + ES-Exos vs. Dox + ESCs, one way ANOVA followed by a Tukey test, Western blot quantities are expressed as A.U. Scale bar = 100 µm**,** *n* = 5–6 for immunohistochemistry (IHC), *n* = 4–5 for Western blot.

Differentiation of monocytes into M1 and M2 macrophages under pathological conditions has been well established [32,33,34]. To investigate whether ES-Exos treatment affect pro-inflammatory M1 Macrophages, IHC staining was performed for iNOS. Significantly (*p* < 0.05) increased expression of iNOS positive cells was observed in the Dox treated group (f–j) versus control animals (a–e), whereas a significant (*p* < 0.05) reduction in the number of iNOS positive cells was observed upon ESCs or ES-Exos treatment (Figure 6A,B). To compliment this data, we examined the presence of TNF-α, a previously reported pro-inflammatory cytokine [32,35]. IHC staining revealed a statistically significant (*p* < 0.05) increase in TNF-α^+ve^ cardiomyocytes (Figure 6C,D) in the Dox group (f–j) as compared with control (a–e), corroborating iNOS data. ESCs (k–o) or ES-Exos (p–t) treatment also showed significantly (*p* < 0.05) reduced TNF-α positive cells as expected in comparison to the Dox group (Figure 6C,D). Moreover, to confirm IHC data for TNF-α, western blot was performed. Densitometric analysis showed a significant (*p* < 0.05) increase in TNF-α expression following Dox administration, however co-treatment with ES-Exos reduced this protein expression significantly (*p* < 0.05, Figure 6E).

**Figure 7:**
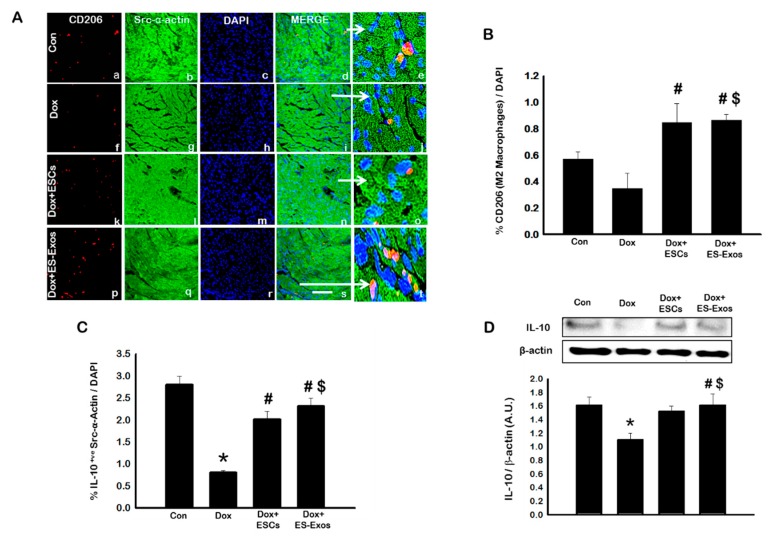
ESCs or ES-Exos enhance M2 macrophages presence and IL-10 expression. (**A**) Representative photomicrographs of heart sections stained with src-α-actin and CD206. Individual boxes show CD206^+ve^ cells in red (a, f, k, p), cardiomyocytes in green (b, g, l, q), DAPI in blue (c, h, m, r), merged images (d, i, n, s), and enlarged areas of merged images (e, j, o, t). (**B**) Quantitative analysis-derived histogram shown for CD206, an indicator marker of M1 macrophages, and (**C**) bar graph derived from quantitative analysis for IL-10^+ve^ cardiomyocytes. (**D**) Representative blot and densitometric analysis shown for IL-10. Error bars = mean ± S.E.M. * *p* < 0.05 vs. control, ^#^ *p* < 0.05 vs. Dox, ^$^ *p* = non significant (NS) Dox + ES-Exos vs. Dox + ESCs, one way ANOVA followed by a Tukey test, western blot quantities are expressed as A.U. Scale bar = 100 µm, *n* = 5–6 for IHC, *n* = 4–5 for Western blot.

Next, we performed analysis for anti-inflammatory M2 macrophage abundance using CD206 immunostaining. Our staining data shows a reduction of M2 macrophages in Dox administered group (Figure 7A, f–j) as compared to control (Figure 7A, a–e), however, data was not statistically significant. In comparison, ESCs or ES-Exos treatment significantly (*p* < 0.05) increased the number of CD206 positive cells (Figure 7B) compared to Dox alone. To verify the increased anti-inflammatory actions upon ES-Exos treatment, we performed IHC staining for IL-10, an anti-inflammatory interleukin. Quantitative analysis showed a significant (*p* < 0.05) decrease in IL-10^+ve^ cardiomyocytes (Figure 7C) in the Dox group as compared with control, suggesting decreased M2 macrophages may correlate with decreased IL-10 following Dox treatment. Dox co-treatment with ESCs or ES-Exos significantly (*p* < 0.05) increased the number IL-10 positive cardiomyocytes compared to the Dox group (Figure 7C). Furthermore, western blot analysis corroborated with IL-10 IHC data, showing a significant (*p* < 0.05) reduction in Dox group, whereas ES-Exos treatment significantly (*p* < 0.05) upregulated IL-10 expression (Figure 7D).

Altogether, these results suggest that ESCs or ES-Exos treatment reduces pro-inflammatory M1 macrophages and pro-inflammatory cytokine TNF-α. In addition, this decrease in pro-inflammatory M1 macrophages occurs with simultaneous stimulation of anti-inflammatory M2 macrophages and IL-10 release, suggesting infiltrated monocytes differentiate into M2 macrophages with ESCs or ES-Exos treatment and create an anti-inflammatory microenvironment in the heart that decreases inflammation and pyroptosis.

### 3.5. Effects of ESCs or ES-Exos Treatment on Mitogen-Activated Protein Kinase (MAPK) Cell Signaling

**Figure 8:**
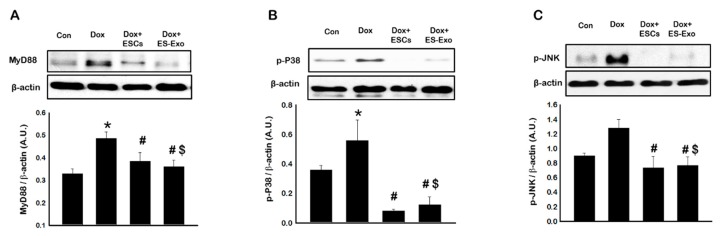
Treatment with ESCs or ES-Exos downregulates mitogen-activated protein kinase (MAPK) signaling pathway in the DIC mouse model. (**A**–**C**) Representative blots and densitometric analysis are shown for (**A**) MyD88, (**B**) p-P38, and (**C**) p-JNK. Error bars = mean ± S.E.M. * *p* < 0.05 vs. control, ^#^ *p* < 0.05 vs. Dox, ^$^ *p* = non significant (NS) Dox + ES-Exos vs. Dox + ESCs, one way ANOVA followed by a Tukey test, Western blot quantities are expressed as A.U. *n* = 5–6.

Current literature suggests that under inflammatory conditions, there is upregulation of cell signaling proteins MyD88, P38, and JNK [36,37]. The upregulation of these cell signaling proteins in Dox treated hearts with and without ESCs or ES-Exos remains unknown. To address these questions, western blot was performed. Our data suggests significantly (*p* < 0.05) increased MyD88 (Figure 8A), p-P38 (Figure 8B), and p-JNK (Figure 8C) in the Dox group compared to control animals. However, this significant increase in cell signaling proteins was reduced following ESCs or the ES-Exos treatment (Figure 8). Our data suggests that (1) Dox-induced pro-inflammatory microenvironment in the Dox treated hearts involves cell signaling proteins MyD88, p-P38, and p-JNK, (2) the induction of pyroptosis within the heart may include these cell signaling proteins, and (3) this process is mitigated with co-treatment of Dox with ESCs or ES-Exos.

### 3.6. Effects of ESCs or ES-Exos on Cytoplasmic Vacuolization, Myofibril Loss, and Cardiac Hypertrophy

**Figure 9:**
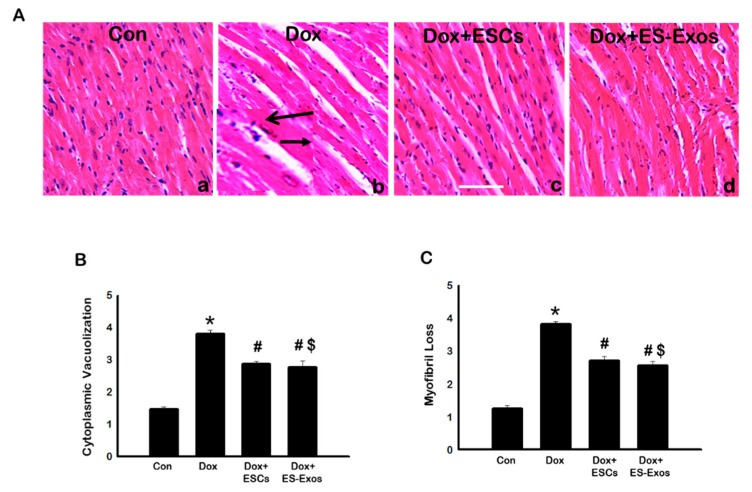
ESCs or ES-Exos inhibit vacuolization and muscle cell loss post-Dox administration. (**A**) Representative images of hematoxylin and eosin (H&E) stained heart sections for all treatment group are shown. (**B**) Quantitative analysis for cytoplasmic vacuolization. (**C**) Quantitative analysis for myofibril loss. Error bars = mean ± S.E.M. * *p* < 0.05 vs. control, ^#^ *p* < 0.05 vs. Dox, ^$^ *p* = non significant (NS) Dox + ES-Exos vs. Dox + ESCs, one way ANOVA followed by a Tukey test, scale bar = 100 µm, *n* = 6–7. Images taken at 40×.

It has already been published that Dox can cause significant structural changes to myocardium including cytoplasmic vacuolization, myofibril loss, and cardiac hypertrophy [24,25,29]. To assess the impact of ESCs or ES-Exos on cardiac remodeling and structural changes in DIC heart, we stained heart sections with hematoxylin and eosin (H&E). Our data shows cytoplasmic vacuolization and myofibril loss was significantly (*p* < 0.05) increased in the Dox group compared to controls (Figure 9B,C, respectively), suggesting the heart has developed cardiac dysfunction. In comparison, ESCs or the ES-Exos treatment significantly reduced the vacuolization and myofibril loss (Figure 9B,C, respectively, *p* < 0.05).

**Figure 10:**
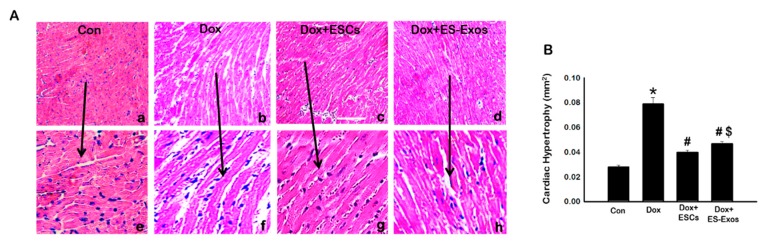
ESCs or ES-Exos protects the heart from Dox-induced hypertrophy. (**A**) Representative cardiomyocyte photomicrographs of H&E stained heart sections for all treatment groups. (**B**) Quantitative analysis for cardiac hypertrophy. Error bars = mean ± S.E.M. * *p* < 0.05 vs. control, ^#^ *p* < 0.05 vs. Dox, ^$^ *p* = non significant (NS) Dox + ES-Exos vs. Dox + ESCs, one way ANOVA followed by a Tukey test, scale bar = 100 µm, *n* = 5–6. Images taken at 20×.

Published studies suggest that there is an increase in cardiomyocyte size during cardiac remodeling following Dox treatment [11,38]. Hypertrophy is a cardioprotective, adaptive mechanism characterized by thickening of the heart muscle accompanied by subsequent diminished cardiac output. To evaluate whether ES-Exos treatment reduces hypertrophy of cardiomyocytes in the DIC heart, H&E stained slides were further analyzed (Figure 10). The cardiomyocyte area was significantly (*p* < 0.05) increased in the Dox group compared to the controls (Figure 10B). In contrast, mice treated with ESCs or ES-Exos showed cardiomyocytes significantly (*p* < 0.05) smaller in size compared with Dox administered mice (Figure 10B). Overall, this data suggest that ESCs or ES-Exos significantly reduced the Dox induced cytoplasmic vacuolization, myofibril loss, and cardiac hypertrophy.

### 3.7. Effects of ESCs or ES-Exos on Fibrosis and Pro-Fibrotic Protein MMP-9

**Figure 11:**
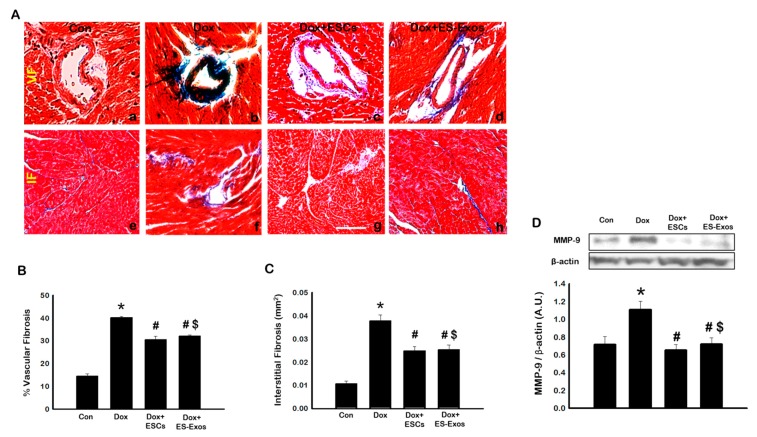
Fibrosis is inhibited with ESCs or ES-Exos treatment post DIC. (**A**) Representative Masson’s trichrome images demonstrating vascular (a–d) and interstitial fibrosis (e–h). (**B**) Percentage of vascular fibrosis quantified over the vessel area. (**C**) Quantitative analysis for intestinal fibrosis. (**D**) Representative blot and densitometric analysis for MMP-9. Error bars = mean ± S.E.M. * *p* < 0.05 vs. control, ^#^ *p* < 0.05 vs. Dox, one way ANOVA followed by a Tukey test, Western blot quantities are represented as A.U. * *p* < 0.05 vs. control. ^#^ *p* < 0.05 vs. Dox, ^$^ *p* = non significant (NS) Dox + ES-Exos vs. Dox + ESCs, scale bar = 100 µm, *n* = 5–6. Images taken at 20×.

Fibrosis is a widely accepted adverse remodeling mechanism that occurs following Dox treatment [6,19]. Masson’s trichrome staining was performed to quantify the collagen deposition during cardiac remodeling. Representative photomicrographs Figure 11A shows vascular fibrosis (a–d) and interstitial fibrosis (e–h) in control mice, Dox treated animals, and treated groups with ESCs or ES-Exos. The percentage of fibrosis was significantly (*p* < 0.05) increased in Dox administered hearts as compared to control, however, the animals treated with ESCs or ES-Exos significantly (*p* < 0.05) reduced both vascular and interstitial fibrosis (Figure 11B,C, respectively).

MMP-9 has been considered as a potential marker for extracellular matrix (ECM) degradation that is associated with fibrosis [11,39]. Therefore, we examined expression of MMP-9 to confirm fibrosis. Our western blot data shows a significant (*p* < 0.05) increase in MMP-9 expression in Dox treated animals as compared to control (Figure 11D). Following treatment with ESCs or ES-Exos the expression level of MMP-9 reduced significantly, (*p* < 0.05) compared with Dox group (Figure 11D). These results suggest that ESCs or ES-Exos treatment decreases both vascular and interstitial fibrosis as well as extracellular pro-fibrotic protein MMP-9 in the Dox-induced cardiomyopathy heart.

### 3.8. ES-Exos Improves Heart Function in DIC Murine Model

**Figure 12:**
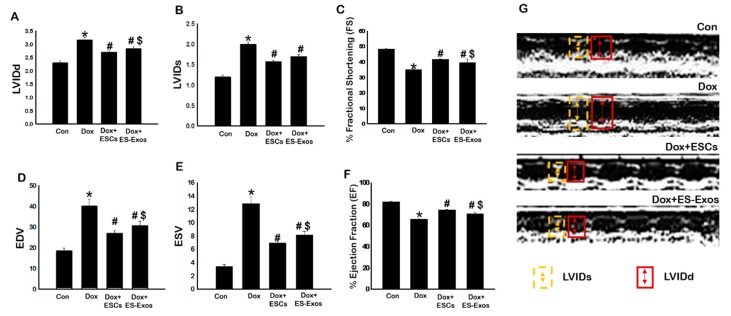
Treatment with ESCs or ES-Exos improves cardiac function following DIC. Two weeks post DIC, 2D transthoracic echocardiography was performed on control and experimental animals. (**A**) Left ventricular internal dimension-diastole (LVIDd), (**B**) LVID-systole (LVIDs), (**C**) fractional shortening (FS), (**D**) end diastolic volume (EDV), (**E**) end systolic volume (ESV), and (**F**) ejection fraction (EF) were analyzed and quantified. (**G**) M-Mode acquisition pictures. Error bars = mean ± S.E.M. * *p* < 0.05 vs. control, ^#^ *p* < 0.05 vs. Dox, ^$^ *p* = non significant (NS) Dox + ES-Exos vs. Dox + ESCs, one way ANOVA followed by a Tukey test, *n* = 6–7. Yellow arrow: LVIDs, red arrow: LVIDd.

To determine the impact of ES-Exos on heart function, 2D transthoracic echocardiography was performed on D14 to evaluate various cardiovascular parameters, including LVIDd, LVIDs, EDV, ESV, FS (measurement of contractility), and EF (measurement of blood ejection from the ventricle) on all control and experimental mice. Analysis of echocardiography data revealed that Dox administered mice have significantly (*p* < 0.05) high values of LVIDd (Figure 12A), LVIDs (Figure 12B), EDV (Figure 12D), and ESV (Figure 12E) compared to the control group. Interestingly, animals treated with ESCs or ES-Exos demonstrated significantly (*p* < 0.05) improved cardiac function as compared to the Dox group, which was measured with LVIDd, LVIDs, EDV, and ESV. FS as an indicator of cardiac contractility and EF that measures left ventricular (LV) blood ejection in the heart were significantly (*p* < 0.05) reduced in Dox treated animals demonstrating decreased cardiac function (Figure 12C,F, respectively). This decreased FS and EF was improved significantly (*p* < 0.05) in the animal treated with ESCs or ES-Exos.

Note: We have performed statistical analysis in all the experiments to understand whether ESCs vs. ES-Exos are statistically different. We did not observe any statistically significant difference between ESCs and ES-Exos and data is reported in the Figures and Figure legends respectively.

## 4. Discussion

Stem cells have been widely used to treat various animal and human cardiac disorders [19,40,41]. Most animal studies associated with stem cell therapy have successfully demonstrated significant repair, regeneration, and cardiac function improvement [19,42,43]. Bone marrow-derived mesenchymal stem cells or embryonic stem cell-derived cardiomyocytes have been shown to repair injured myocardial infarcted heart, ischemic reperfusion injury, and Dox-induced heart failure in mice and rabbits [19,44,45,46]. Adult stem cell clinical studies in patients suffering from heart failure have shown mixed responses on the improvement of heart function [19,47]. In addition, ESC-derived cardiomyocytes or vascular smooth muscle cells have been shown to be an optimal cell type for cardiac cell therapy compared with adult stem cells [19,48]. However, the hope for ESC therapy was hampered when published reports showed potential chances of tumor formation [20,49,50].

Recent discovery of a cell-free system using Exos have generated a new era in cell therapy for cardiac repair and regeneration [23,28]. Current reports suggest that derived Exos from ESCs can regenerate infarcted heart, enhance myocardial viability and attenuate adverse cardiac remodeling in ischemic conditions [28]. Similarly, isolated Exos from adult stem cells, cardiac progenitor cells, and cardiac stem cells have shown protective effects against ischemic myocardium from apoptosis and adverse cardiac remodeling [1,51,52,53,54]. However, it remains unknown whether Exos isolated from ESCs have a potential to inhibit (1) inflammation-induced cell death: Pyroptosis, (2) attenuate inflammatory M1 macrophage abundance, (3) enhance anti-inflammatory M2 macrophage population, and (4) reduce Dox-induced adverse cardiac remodeling including cytoplasmic vacuolization, myofibril loss, cardiac hypertrophy, and fibrosis. We further evaluated the effects of these ES-Exos on cardiac dysfunction induced by Dox.

We recently reported in our cell culture system that Dox induces TLR4-NLRP3 mediated pyroptosis in H9c2 cells [1]; however, this remains unknown whether pyroptosis occurs in an in vivo model of DIC. The current study investigates the presence of pyroptosis in DIC in vivo, focuses on understanding the effects of transplanted ES-Exos and compares the beneficial effects of ES-Exos vs. ESCs. Pyroptosis is an inflammation-mediated cell death that is believed to occur when there is a bacterial infection initiating inflammation [55]. Recent studies show that *Salmonella* and *Shigella* species caused caspase-1 mediated pyroptosis in macrophages that results in the release of IL-1β and IL-18 [55,56,57].

However, published study reports that NLRP3 inflammasome-mediated pyroptosis occurs in diabetic rats following ischemic reperfusion injury within the heart where pyroptosis was not initiated by a bacterial infection [58]. Therefore, it is hypothesized that pyroptosis present in non-infectious tissues such as heart is initiated through DAMPs (damage-associated molecular pattern molecules) termed as sterile inflammation. The sterile inflammation-induced pyroptosis is initiated through activation of the TLR4 receptor, which results in the formation of the NLRP3 inflammasome as published previously [59,60,61]. This response induces the activation of the caspase-1 cascade and causes downstream secretion of pro-inflammatory cytokines IL-1β and IL-18 as observed in diabetic infarcted hearts [31,60,61]. In the present study, a DIC model of pyroptosis was generated in C57BL/6J mice and was confirmed via the upregulated presence of inflammasome markers (TLR4 and NLRP3). Furthermore, we examined presence of pyroptotic markers caspase-1, IL-1β, and IL-18 using immunohistochemistry and western blot analysis that indicated significant (*p* < 0.05) upregulation of these markers and confirmed pyroptosis as reported by various investigators [31,58,60,62]. Moreover, ES-Exos used in the present study inhibits TLR4 and NLRP3 inflammasome markers and inflammasome-induced pyroptosis in DIC. These in vivo data in DIC are in conjunction with our previously published reports on ES-Exos demonstrating inhibition of the inflammasome and pyroptotic proteins in Dox-induced pyroptosis in H9c2 [1] and Sol 8 [29] cells.

Next, we examined the presence of pro and anti-inflammatory cytokines as well as hypothesized whether Dox-induced cardiomyocyte apoptosis or necrosis cause infiltration of monocytes to clear dying cardiomyocytes, which were unable to be cleared naturally. Our data confirmed presence of significantly increased M1 macrophages in DIC heart and generated a pro-inflammatory microenvironment, which was further confirmed with secretion of pro-inflammatory cytokine, TNF-α. Whether this increased M1 macrophage level might have caused inflammation-mediated pyroptosis in DIC hearts remains unknown and requires further investigation.

Interestingly, ES-Exos enhanced anti-inflammatory M2 macrophages in DIC, which is in corroboration with other previously published literature on the effects of Exos on macrophage polarization [63,64,65]. Moreover, this increased M2 macrophage level created an anti-inflammatory microenvironment, which was confirmed with the presence of anti-inflammatory cytokine IL-10. The transplanted ES-Exos also inhibited inflammasome and pyroptotic markers compared with Dox-treated hearts as mentioned above. Therefore, these findings raises an important question about the specific contents of Exos including miRNA, growth factors, proteins, and anti-inflammatory cytokines that may play a role in converting M1 into M2 macrophages or directly inhibiting pyroptosis in DIC heart. Our recently published data showed ES-Exos predominantly enriched with anti-inflammatory cytokines [1]. However, this remains unknown whether these anti-inflammatory cytokines in these ES-Exos inhibit pyroptosis or enhance M2 macrophages specifically.

Moreover, we investigated the possible cell signaling pathway involved in pyroptosis in DIC hearts with and without ES-Exos treatment. Our data shows pro-inflammatory cell signaling proteins MyD88, p-P38, and p-JNK following inflammation were significantly increased in DIC, which is in agreement with previously published studies [66,67,68]. Following treatment with ESCs or ES-Exos, we observed for the first time a reduction in cell signaling proteins, which might have played a role in decreased pyroptosis in DIC.

According to recently published literature, DIC heart shows cytoplasmic vacuolization, myofibril loss, and hypertrophy of cardiomyocytes (hallmarks of cardiomyopathy), where these alterations preserve the architecture of the heart [11,19]. However, these changes further enhance cardiac fibrosis involving MMPs-induced ECM degradation and leads to cardiac dysfunction [6,11,19]. Therefore, we hypothesized whether treatment with ES-Exos can inhibit cytoplasmic vacuolization, myofibril loss, and hypertrophy as well as cardiac fibrosis, a major contributor in DIC leading to heart failure. To the best of our knowledge, there are no published studies demonstrating that ES-Exos can preserve cardiac architecture by inhibiting cytoplasmic vacuolization, myofibril loss, hypertrophy, and fibrosis in DIC hearts. The observed vascular fibrosis in DIC heart was intense in the vascular and peripheral area of the vessels whereas interstitial fibrosis in these hearts was sporadic (Figure 11). This significant increase in fibrosis was inhibited by ES-Exos. Furthermore, we observed significant reduction in ECM degradation protein, MMP9. Therefore, this set of data concludes that ES-Exos has the potential to inhibit adverse cardiac remodeling in DIC hearts. However, the mechanisms of inhibition of adverse cardiac remodeling with ES-Exos remain elusive.

After inhibition of inflammation-induced pyroptosis and adverse cardiac remodeling following ES-Exos transplantation, we determined whether these Exos have any effects on cardiac dysfunction observed following Dox treatment. Interestingly, our data shows ES-Exos significantly (*p* < 0.05) improved Dox-induced cardiac dysfunction (Figure 12).

Moreover, recent studies suggest that intraperitoneally (i.p.) transplanted stem cells or derived exosomes showed protection in cardiac and many other diseases [19,69,70,71,72,73] The current study uses i.p. injections to administer ESCs or ES-Exos and shows inhibition of pyroptosis and adverse cardiac remodeling in DIC. Therefore, our data corroborates with the previously published studies as stated above that used i.p injection to administer stem cells or derived exosomes. However, this would be interesting and open a new door to inject ESCs or ES-Exos using intravenous (i.v.) injections to determine whether i.v. injections have different protective effects on cardiac remodeling in DIC.

**Figure 13:**
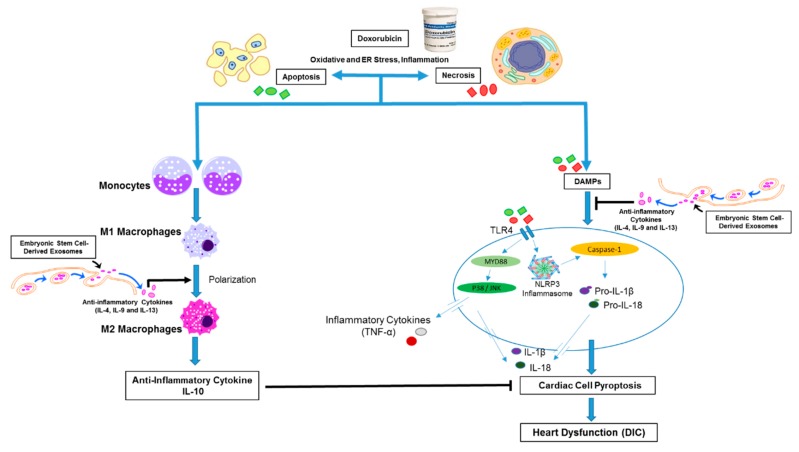
Treatment with ES-Exos reduces Dox-induced heart dysfunction. Schematic picture represents that Dox induces pyroptosis and increases inflammation via enhanced M1 macrophage population. However, treatment with ES-Exos can attenuate pyroptosis, increase M2 macrophage polarization and anti-inflammatory cytokine release (IL-10), which is potentially mediated through some anti-inflammatory cytokines present in ES-Exos. ER; endoplasmic reticulum, DAMPs; damage-associated molecular pattern molecules, TLR4; toll-like receptor 4, NLRP3; nucleotide-binding oligomerization domain-like receptor pyrin domain containing-3.

In conclusion, the current study provided evidence that Dox induced pyroptosis and inflammation, which could be inhibited upon treatment with ES-Exos leading to overall preservation of cardiac function, presented in schematic diagram (Figure 13). This was a novel study with new mechanistic observations on the pathophysiological role of pyroptosis in Dox-induced cardiotoxicity. Additionally, this study emphasized whether ES-Exos had equal potential compared with ESCs used in cell therapy. Our data on ES-Exos and ESCs shows inhibition of pyroptosis, cell signaling, adverse cardiac remodeling, and improved heart function with equal in quality and quantity. Therefore, this cell-free system, ES-Exos, could be a future therapeutic agent in clinical investigations without losing any potential benefits compared to ESCs where we had no further threat of teratoma formation.

## Figures and Tables

**Table 1:** Primer sequences used for the study.

Target	Forward Primer	Reverse Primer
GAPDH	5′-ACCCAGAAGACTGTGGATGG-3′	5′-CACATTGGGGGTAGGAACAC-3′
Caspase-1	5′-GAAACGCCATGGCTGACAAG-3′	5′-CGTGCCTTGTCCATAGCAGT-3′
IL-1β	5′-AACCTGCTGGTGTGTGACTTC-3′	5′-CAGCACGAGGCTTTTTTGT-3′
IL-18	5′-ACTTTGGCCGACTTCACTGT-3′	5′-GTCTGGTCTGGGGTTCACTG-3′

## Abbreviations

ANOVA	analysis of variance
BW	body weight
DAPI	4′,6-diamino-2-phenylindole
DIC	doxorubicin-induced cardiotoxicity
DMEM	dulbecco’s modified eagle’s medium
Dox	doxorubicin
EDV	end diastolic volume
EF	ejection fraction
ER	endoplasmic reticulum
ESCs	embryonic stem cells
ESV	end systolic volume
Exos	exosomes
FBS	fetal bovine serum
FS	fractional shortening
H&E	hematoxylin and eosin
HW	heart weight
IHC	immunohistochemistry
IL	interleukin
iNOS	induced nitric oxide synthase
IP	intraperitoneal
LVIDd	left ventricular internal dimension-diastole
LVIDs	left ventricular internal dimension-systole
MI	myocardial infarction
miRNA	microRNA
MMP	matrix metalloproteinase
PBS	phosphate buffered saline
PS	penicillin/streptomycin
PVDF	polyvinylidene difluoride
RIPA	radio-immunoprecipitation assay
RT-PCR	real time-polymerase chain reaction
Src-α-actin	sarcomeric alpha actin
SDS	sodium dodecyl sulfate
SEM	standard error of mean
